# Parental acceptance of novel children's medical syringes and their influencing factors

**DOI:** 10.3389/fpsyg.2025.1454108

**Published:** 2025-03-11

**Authors:** Su Wen Luo, Peng Rui Yang

**Affiliations:** Faculty of Art and Design, Hubei University, Wuhan, China

**Keywords:** children medical supplies, TAM modeling, design decisions, user attitude, design model

## Abstract

**Background:**

With the rising global demand for medical syringes among children, the unsuitability of traditional syringes may negatively affect their physical and mental health.

**Methods:**

This study integrates the extended Technology Acceptance Model (TAM) to survey 455 child guardians on 10 variables influencing their attitudes toward pediatric medical syringes.

**Results:**

Results indicate that aesthetic preferences of users significantly influence the price value sensitivity and purchasing decisions of children's guardians. Furthermore, the product's function and price value significantly shape users' behavioral intentions. Technology anxiety and time and error reduction emerge as key factors influencing perceived risks.

**Conclusions:**

This study offers product designers crucial insights into purchasing factors for children's medical products, aims to enhance product development and iteration efficiency, and promotes more accurate innovation, decision-making, and communication. Additionally, it proposes new recommendations for ethical and marketing strategies.

## Highlights

- Based on a survey of 455 children's caregivers, this study found that aesthetic preference, price value, functionality, and perceived risk are important variables influencing users' attitudes and purchase decisions of children's medical products.
- This study fills the research gap on the parents toward children's medical products and their decision-making factors, and bridges the gap between theoretical models and practical applications in the design and marketing of children's medical products.
- This research has significantly influenced and improved design innovations, decision-making processes, and marketing strategies for pediatric medical products, increasing caregiver acceptance and satisfaction, leading to better health outcomes for children.

## 1 Introduction

People's lifestyles and production methods have changed dramatically as a result of advances in information and medical technology. Furthermore, consumer needs and product expectations are continually evolving. Consequently, designers and companies must refine their approaches and optimize the design decision-making process to more effectively meet consumer needs for products. Medical products are indispensable for children's development. The use of medical products differs from everyday products as they are necessitated rather than desired (Evode et al., 2021).

Considering their unique developmental context, children represent a specialized group with health and social needs distinct from those of adults (Jack and Phoenix, 2022). For instance, medical products designed for children necessitate ergonomic designs tailored to their size and materials that ensure enhanced safety compared to those for adults. As a result, adults' understanding of children's needs does not always coincide with the perspectives or priorities of the children themselves (Krutzinna, 2022). Although safety, affordability, ease of installation and maintenance, durability, and functionality are crucial for health service products, the absence of child-friendly and aesthetically appealing designs can compromise the overall quality of the children's experience (Manouchehri et al., 2021). Children value supportive environments (Meng et al., 2025), products specifically designed for them (World Health Organization and UNICEF, 2022), age-appropriate spaces (Grace et al., 2023), aesthetic appeal (Anvarjonovna, 2021), and a distinct preference for color (Thung and Ahmad, 2022), among other aspects.

In the Chinese market, the commercialization of children's medical products is still in its early stages. Due to traditional perspectives, consumers often exhibit conservative attitudes and express uncertainty regarding these products. Given that buyers are usually parents or caregivers, their attitudes and behavioral intentions toward children's medical products are crucial, playing a decisive role in the development and optimization of product strategies. Consequently, conducting studies, especially those exploring consumer attitudes through the lens of technology adoption and usage processes, becomes imperative to help caregivers overcome their apprehensions about new technologies and enhance their attitudes toward usage.

Nonetheless, limited research on the barriers to and limitations of accepting medical products, particularly in children's medical product design, scant attention has focused on the attitudes of actual purchasers—parents—toward these products and the primary factors influencing their decisions. Caregivers are more than just purchasers; They are able to gain insight into the needs and preferences of the child, providing valuable insights. Investigating these factors is crucial for driving innovation in the industry and enhancing customer needs and services. These results could potentially affect parents' future acceptance of pediatric medical products. Understanding users' behavior and relationship with new technologies necessitates an understanding of the factors driving their adoption or rejection.

The potential determinants and pathways influencing parents' use of children's medical products are therefore explored in this study. Using a children's medical syringe as a case study, we developed a research hypothesis through a survey to investigate the factors influencing guardian acceptance of children's medical products. Utilizing data from product conceptualization to user case surveys, this study employs a unified model of the theory of the acceptance and use of technology (Venkatesh et al., 2012), incorporating variables such as technology anxiety (TAX), time and error reduction (RTE), price-value (PV), functionality (FUN), aesthetics (AES), perceived usefulness (PU), perceived ease of use (PEOU), perceived risk (PR), attitude toward use (ATU), and behavioral intentions (BI). Additionally, the model includes variables such as age, gender, and frequency of use to examine their moderating effects on the model's structure, utilizing structural equation modeling (SEM) for analysis.

The consequences of this research enhance the understanding of caregiver acceptance of children's medical supplies and have significant implications for advancing product design and innovation, crucial for both the Chinese and global markets. This study is anticipated to make a substantial contribution to the development and maturation of the children's medical product market in China.

## 2 Theoretical background

### 2.1 Child medical product

A wide variety and substantial number of medical products, as defined by China's Regulations for the Supervision and Administration of Medical Devices, exist. These devices encompass instruments, equipment, apparatus, materials, and other articles, including necessary software, used singly or in combination, within the human body (Bernard et al., 2018). Furthermore, a vast array of medical products specifically designed for children is available. Surveys and analyses of relevant literature reveal that existing children's medical products can be classified into four major functional categories: diagnostic, therapeutic, auxiliary, and rehabilitation training equipment.

As the child population increases, the demand for children's products has significantly escalated. Researchers have explored various factors affecting the design and adoption of children's medical products, including ergonomics, entertainment needs, color sensitivity, and material safety requirements. For instance, Goto et al. (2021) investigated the 3D head and face anthropometrics of children, examining its influence on product dimensions and shape alterations during development. Ingadottir et al. (2022) adopted a user-centered, formative mixed-methods approach, utilizing games to develop and test usability, Gao (2021) employed big data and machine learning to analyze children's color preferences across age groups and parents' concerns about materials and functionalities in children's products.

The literature review indicates that numerous studies have focused on children's attitudes toward products, their evaluations, and the integration of their needs in the design process. For example, Parbhu et al. (2019) explored the perceptions of children, parents/caregivers, and nurses regarding new and existing IV pole designs; Gennari et al. (2017) assessed the 8–10-year-olds' performance in game design through participatory play. Additionally, other studies have examined stakeholders and usage contexts in pediatric healthcare (Høiseth et al., 2014), as well as the connection between parents' habits and children's health (Eymirli et al., 2021).

Current research and development shows that these trends are focused on patient comfort, safety, and efficiency, reflecting continued progress and innovation in the design and use of relevant pediatric medical devices.

### 2.2 Technology acceptance model (TAM)

The TAM Model is primarily used to evaluate technology acceptance levels. Researchers such as Davis (1985), Venkatesh et al. (2003), and others have applied theories of human behavior to analyze users' inclinations toward technology adoption. Initially used to explore the factors behind the widespread acceptance of computers, TAM has become a major model for analyzing user behavior (Davis, 1989). This model investigates diverse factors that shape behavioral intentions and actual use of technology, leading to user acceptance.

**Figure 1:**
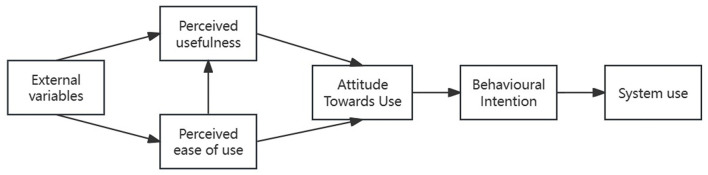
Technology acceptance model (TAM).

The TAM Model is widely recognized as a highly effective tool for elucidating the acceptance and readiness to use information technology (Jacoby, 1983; Davis, 1989). This model elucidates consumer acceptance and corresponding behaviors toward innovative technologies, with a specific focus on information technology. The core elements of the TAM model are PU, PEOU, ATU, and BI as shown in Figure 1 Currently, TAM is extensively applied in research on child-related products and behavioral studies.

Based on TAM, users' PU and PEOU of a new technology influence users' attitudes toward it, affecting their willingness to adopt the technology and ultimately their actual behavior. Technology acceptance is largely determined by the user's expectations and evaluations, making it a personal process (Korkmaz et al., 2022). The individual's experience during use dictates the level of acceptance, making it a personal decision (Schade and Baum, 2007). The relationship between the adoption of new technology and user behavior is clarified by understanding technology acceptance. The core variables of the model include PEOU and PU of the technology. Price value (PV) is also considered a crucial factor in technology acceptance and usage, prompting decision-makers to carefully consider costs before implementation.

## 3 Research model and hypothesis

**Figure 2:**
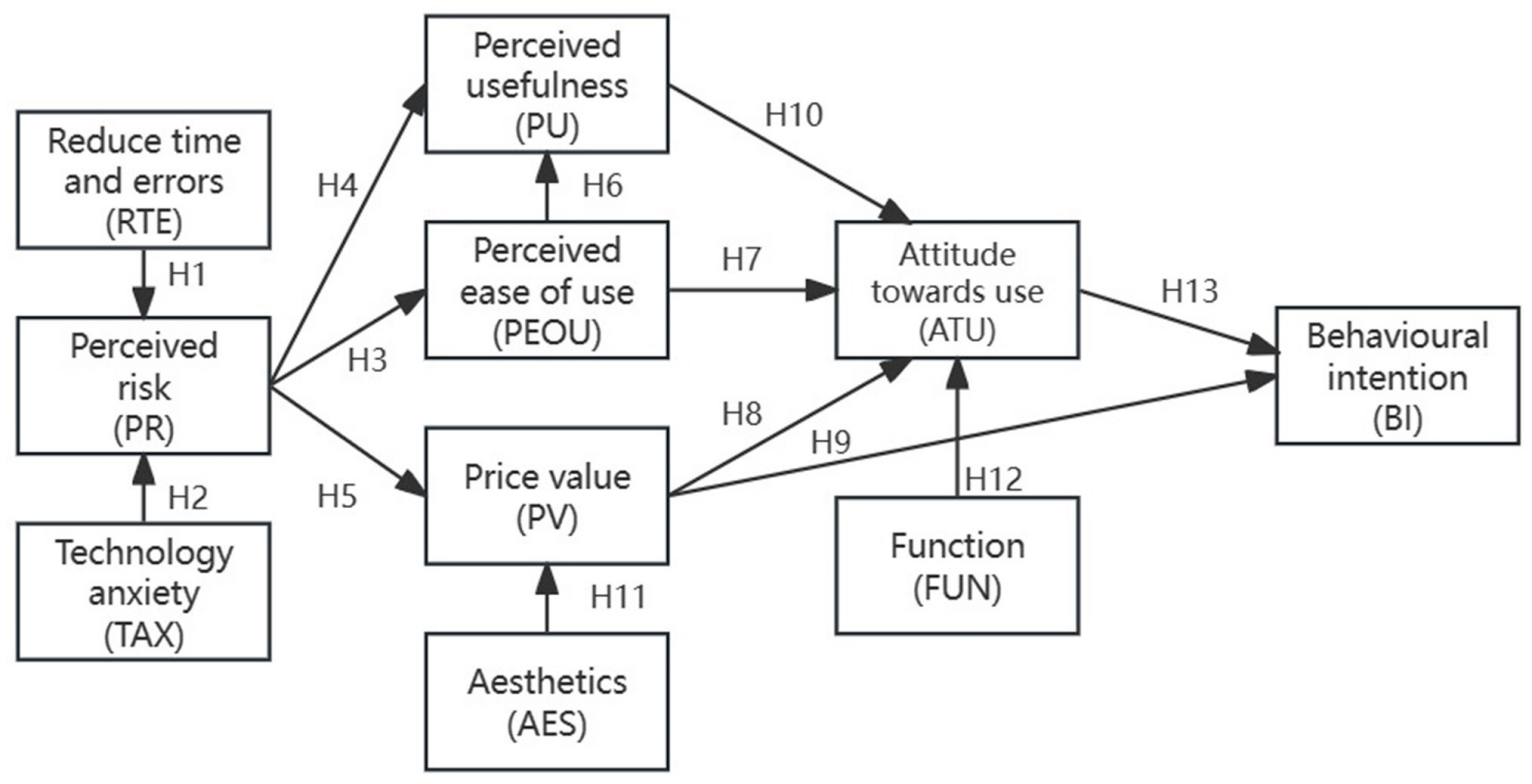
Research model.

This research aims to analyze factors influencing guardians' attitudes (ATU) and behavioral intentions (BI) toward using children's medical products. Consequently, this study extends the traditional TAM model, as illustrated in Figure 2, by incorporating six additional factors that may influence guardians' ambivalent attitudes toward new children's medical products, and 12 research hypothetical paths are proposed. (1) Perceived risk (PR) associated with using the new device (Venkatesh and Davis, 2000; Rehman et al., 2020). (2) Price value (PV) of the product (Lau et al., 2021). (3) Reduction in time and error (RTE) (Albagmi, 2021). (4) Anxiety regarding the new product's technology (TAX) affecting adoption rates (Kamal et al., 2020). (5) Aesthetics (AES) of the product (Riggle, 2024). (6) Functionality (FUN) of the product (Yang et al., 2016; Riggle, 2024). Building on TAM, this study proposes the following hypotheses:

1. Reduction of time and errors (RTE)

In the relm of children's medical products, caregivers typically base their purchase decisions on the technical features of the product. Mitsea et al. (2022) assert that visual information significantly decreases search and reading time, enhances usage efficiency, and lowers error rates. Conversely, Feng (2025) argue that cognitive offloading assists reduce cognitive load and optimize the computational process. In the context of children's medical products, safety and accuracy are pivotal in consumer decision-making.

An increase in RTE signifies a reduced likelihood of product inaccuracies during use, thereby diminishing functional risk (Featherman et al., 2021). As the product's accuracy and effectiveness are confirmed, consumer trust increases, thereby alleviating concerns about its safety and efficacy. Parents frequently worry about the negative consequences of incorrect choices in selecting medical supplies for their children, and an enhancement in RTE can help mitigate this psychological pressure. Having considered the above, we then proceeded to hypothesize the following:

H1: RTE positively influences PR.

2. Technology anxiety (TAX)

Research on innovation indicates that the initial trial of innovation rarely elicits neutral responses, often provoking strong emotional reactions from consumers, including anxiety (Colombo et al., 2021; Rainieri et al., 2023), before its first use. Technology anxiety among consumers may affect their willingness to try new technologies. Chang and Chen (2021) and Cruz-Cárdenas et al. (2021) emphasized the importance of technology readiness for consumers to experiment with new technologies. Henderson and Corry (2021) found that in the technological revolution, users included not only fear of new use of technology, but also pressure and resistance.Technology anxiety significantly increases the perceived risk among users, especially for products like children's medical supplies that require precise handling and substantial trust.

Therefore, reducing users' technology anxiety and increasing their confidence and familiarity with the technology is crucial for lowering perceived risk and enhancing user acceptance. Furthermore, technology anxiety might trigger fears of financial losses owing to technical issues, such as product failure or additional repair costs (Yap et al., 2021). This study explores the relationship between technology anxiety (TAX) and perceived risk (PR). We hypothesize that consumers' attitudes toward technology anxiety may influence their level of perceived risk. Accordingly, we propose the following hypothesis:

H2: TAX positively influences PR.

3. Perceived risk (PR)

PR is the subjective evaluation by consumers or users of potentially unfavorable outcomes associated with a product or service. Featherman et al. (2021) argued that heightened uncertainty in the context of new product adoption can increase perceived risk, serving as a barrier to adoption. Cabeza-Raḿırez et al. (2022) observed that higher levels of perceived risk lead to more pronounced negative attitudes and behavioral intentions. Habibi and Rasoolimanesh (2021) investigated the interplay between client satisfaction and perceived risk in experience and service quality organizations, taking into account customers' previous experiences with the service provider.

Perceived risk is a multidimensional construct that includes safety, financial, social, time, and performance risks (Alrawad et al., 2023). In research, PR is identified as a crucial variable in interpreting satisfaction and intention to use technology. Syrová and Špička (2022) identified management performance and financial risks as the two primary dimensions often considered when assessing perceived risk. Financial risk involves potential monetary losses customers may face, including repair or replacement costs and refund issues.

In the context of Pediatric devices, perceived risks to a child's health and safety are particularly pronounced, requiring focused attention and management. It is assumed that after using a children's medical product, customers may become aware of the potential risks associated with the product. When parents are apprehensive about potential risks associated with a new product, perceived risk (PR) may influence their perceptions of the product's PU and PEOU, subsequently impacting their purchase decisions and usage behavior. Additionally, the financial aspect of perceived risk can influence consumers' purchase decisions, especially when the cost of medical supplies exceeds the household budget or involves additional repair or replacement expenses. Based on this, we propose the following assumptions:

H3: PR positively influences PEOU.

H4: PR positively influences PU.

H5: PR positively influences PV.

4. Perceived ease of use (PEOU)

PEOU represents the user's subjective assessment of the effort required to use a specific technology or system, essentially measuring the ease of technology use. Within the TAM framework, PEOU, along with PU, is a primary element of technology acceptance and use. PEOU reflects the degree to which users perceive that operating a new technological system requires minimal effort, serving as intrinsic motivation (Wilson et al., 2021). Venkatesh and Bala (2008) highlight that technology which is clear and straightforward will be easier to use, enabling users to complete tasks effortlessly.

The significance of PEOU increases in applications for children's medical products, as users like parents and healthcare professionals may lack extensive medical or technical expertise (Razu et al., 2021; Hagström et al., 2022). Therefore, ensuring these products are user-friendly and easy to operate is crucial for enhancing their acceptance and effective use. Based on this understanding, we explore the relationship between PEOU, PU, and consumer attitudes toward children's medical products, leading to the proposal of the following assumptions:

H6: PEOU positively influences PU.

H7: PEOU positively influences consumer attitudes toward the use of children's medical products (ATU).

5. Price value (PV)

Price Value (PV) is a crucial concept in marketing and consumer behavior, representing the customer's assessment the price of a product or service. Patil and Rane (2023) assert that value for money plays a key role in attracting consumers, and that businesses that are able to improve their customer value proposition are more likely to gain users' purchase preferences. In the consumer decision-making process, price value is a pivotal factor in determining a product or service's value for money. Hride et al. (2022) demonstrated that perceived fair pricing has a substantial and a beneficial effect on satisfaction. When the benefits of using a technology outweigh its monetary costs, the impact is perceived positively (Anwar et al., 2021).

In the market for children's medical products, parents' purchasing decisions are significantly influenced by value for money, making the pricing mechanism a crucial factor. When purchasing these products, parents consider factors such as price, safety, efficacy, and brand reputation. An effective pricing strategy can enhance consumer trust and increase sales (Cakranegara et al., 2022; Nagle et al., 2023). Given the importance of their children's health and safety, parents may be willing to pay a premium for high-quality and reliable products. Pricing serves to indicate the balance between perceived benefits and costs associated with using pediatric medical products. Based on the above, we propose to hypothesize as follows:

H8: PV positively influences BI.

6. Perceived usefulness (PU)

Perceived usefulness (PU), a central notion in the Technology Acceptance Model (TAM), involves users' subjective evaluation of how a specific technology, product, or service enhances productivity or quality of life (Siagian et al., 2022). This concept includes the user's perception of benefits derived from using a particular technology or product (Oyman et al., 2022). Venkatesh and Davis (2000) suggested that PU could serve as a metric to gauge a system's suitability for specific tasks and the strength of belief in its capacity to improve personal performance. Therefore, PU is viewed as convenient, beneficial, and conducive to success.

Venkatesh et al. (2003) emphasizes that PU is a crucial predictor of the Readiness to use and embrace technology, significantly influencing consumers' ATU. In the context of children's medical supplies, perceived usefulness is focused on enhancing or safeguarding children's health (National Academies of Sciences, Engineering, and Medicine et al., 2018). Parents or healthcare professionals are more likely to adopt a product if they perceive significant benefits in monitoring and maintaining children's health. For example, a child health monitoring device recommended by a physician may be perceived as more useful by parents. According to the aforementioned research, regarding the relationship between PU and ATU, we formulate the following propositions:

H9: PU positively influences consumer attitudes (ATU) toward the use of children's medical products.

7. Aesthetics (AES)

Aesthetics is rooted in the human visual sensory system and the degree to which the brain adapts to the environment. In product design, aesthetics is concerned with the final finish of the design, shape, or its style. Shi et al. (2021) pointed out that products with good aesthetics are more likely to be accepted by many people. Aesthetics significantly increase the perceived value of a product to consumers. Recent research have shown that parents are often more willing to pay a premium price for visually appealing products that meet aesthetic standards (Xie et al., 2023). In the field of smart products, Bakhshian and Lee (2022) pointed out that aesthetics is a key determinant of product acceptability and wearability.

Aesthetics play a crucial role in shaping consumers' perception of the value of a product (Kirillova and Chan, 2018). Innovative and visually appealing designs can increase market demand for children's medical products, especially for parents. Shaharuddin and Jalil (2021) point out that there is a positive relationship between a product's visual appeal and pricing, with parents willing to pay more for the aesthetic design of a children's product. In the market for children's medical products, attractive and innovative designs can make parents perceive these products as more valuable. Consequently, outstanding aesthetic design can increase the market appeal of children's medical products and may enable manufacturers to set a higher price point. Based on those considerations, we propose the following hypotheses:

H10: AES positively influences PV.

8. Function (FUN)

In product design and marketing, functionality (FUN) refers to the actual functions and features a product or service offers, crucial for meeting consumer needs and solving specific problems (Misischia et al., 2022). Given the diverse needs of the audience, the medical device design for kids should incorporate perspectives from healthcare professionals, parents, and children, thereby strengthening the connection among these groups (Pradhan et al., 2021). Children's medical products need to be designed not only with basic functional principles, but also with enhanced portability and versatility to meet the high demands of contemporary society for smart device connectivity and user interaction.

Moreover, Guerlich et al. (2023) pointed out that children's medical products should pay more attention to functional innovation in user health monitoring, interaction design, and adjuvant therapy, hoping to improve the quality of medical products by improving the user's medical experience. Haleem et al. (2022) further pointed out that in the context of modern scientific and technological progress, the design of children's medical devices should not only focus on the function of the product itself, but also consider its interactive application potential in improving the efficiency of health management and enhancing the information communication between parents and doctors. Based on these insights, we propose the following hypotheses regarding functionality (FUN) variables:

H11: FUN positively influences consumer attitudes (ATU) toward the use of children's medical products.

9. Attitude toward using (ATU)

Attitude delineates an individual's intent to act in specific ways across various contexts (Fishbein, 1967). ATU often linked to attitudes, awareness, and usage studies, is defined as the positive or negative attitudes a user forms toward a system (Fietta et al., 2021). Within the TAM framework, ATU is an intermediate variable influenced by PEOU and PU, affecting the user's behavioral intention (BI).

An individual's behavior is shaped by their motivation to perform said behavior, which is affected by their ATU (Saif et al., 2024). Moon and Kim (2001) and Flanigan (2021) characterized ATU as encompassing variables such as good/bad, smart/stupid, enjoyable/unenjoyable, and positive/negative. For this study, we adopt a positive definition of ATU. These emotional factors play a key role in a variety of application areas, so understanding the role of ATUs in the study of user behavior models is critical to driving user technology adoption and product design.

10. Behavioral intention (BI)

Behavioral intention (BI) indicates a user's willingness to use a system (Humida et al., 2022). Within the TAM framework, BI is a critical outcome variable affected by PU, PEOU, and ATU. Venkatesh and Bala (2008) defined behavioral intention as encompassing the motivation to use, anticipation of use. Understanding consumers' attitudes, perceptions, and usage of children's medical products is vital for predicting their future behavioral intentions. For example, consumers are likely to show a strong intention to use a children's medical product if they perceive it as safe, effective, and reliable (Rahardja et al., 2024). This is mainly because parents and medical professionals tend to be more cautious when choosing medical products for children, and pay more attention to the safety and effectiveness of different dimensions of the products (Chen et al., 2022). Building on these positive attitudes, we have developed the following hypotheses regarding attitudes and behavioral intentions toward children's medical products:

H12: Consumers' attitudes (ATU) toward children's medical products positively influence BI.

## 4 Methods

### 4.1 Children injection product methodology

**Figure 3:**
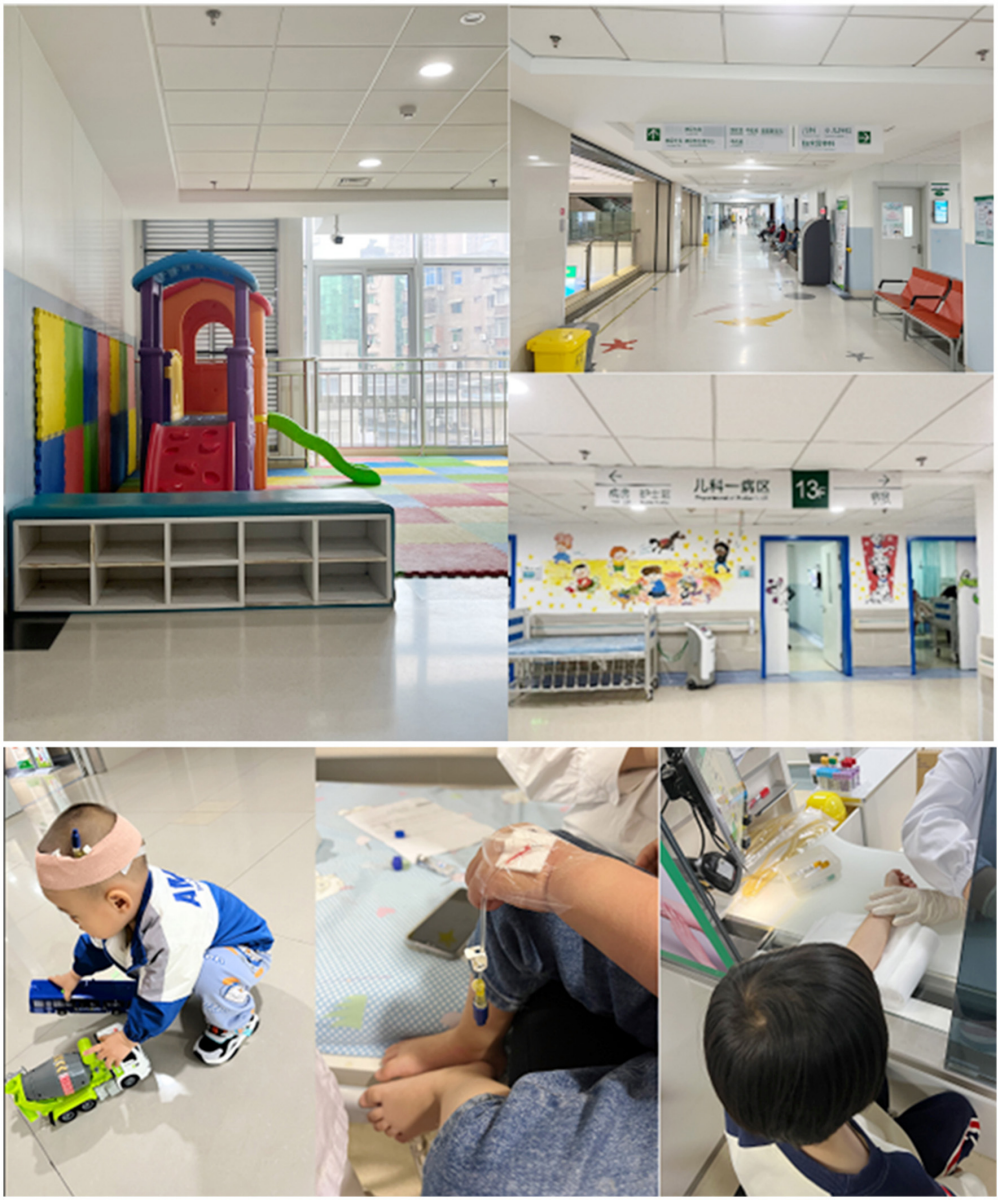
Diagram of the field scene.

Conducting field research in scenarios representative of the research objectives, which include both target users and environments (Wolstenholme, 2016), effectively identifies genuine pain points and opportunities. To design the conceptual model of the children's medical injector, we conducted field research at the Xiling Branch of Yichang City Central Hospital to exemplify the current children's healthcare environment. We observed the facilities of medical institutions and the process of child counseling, and collected relevant information through field observations, as shown in Figure 3.

**Figure 4:**
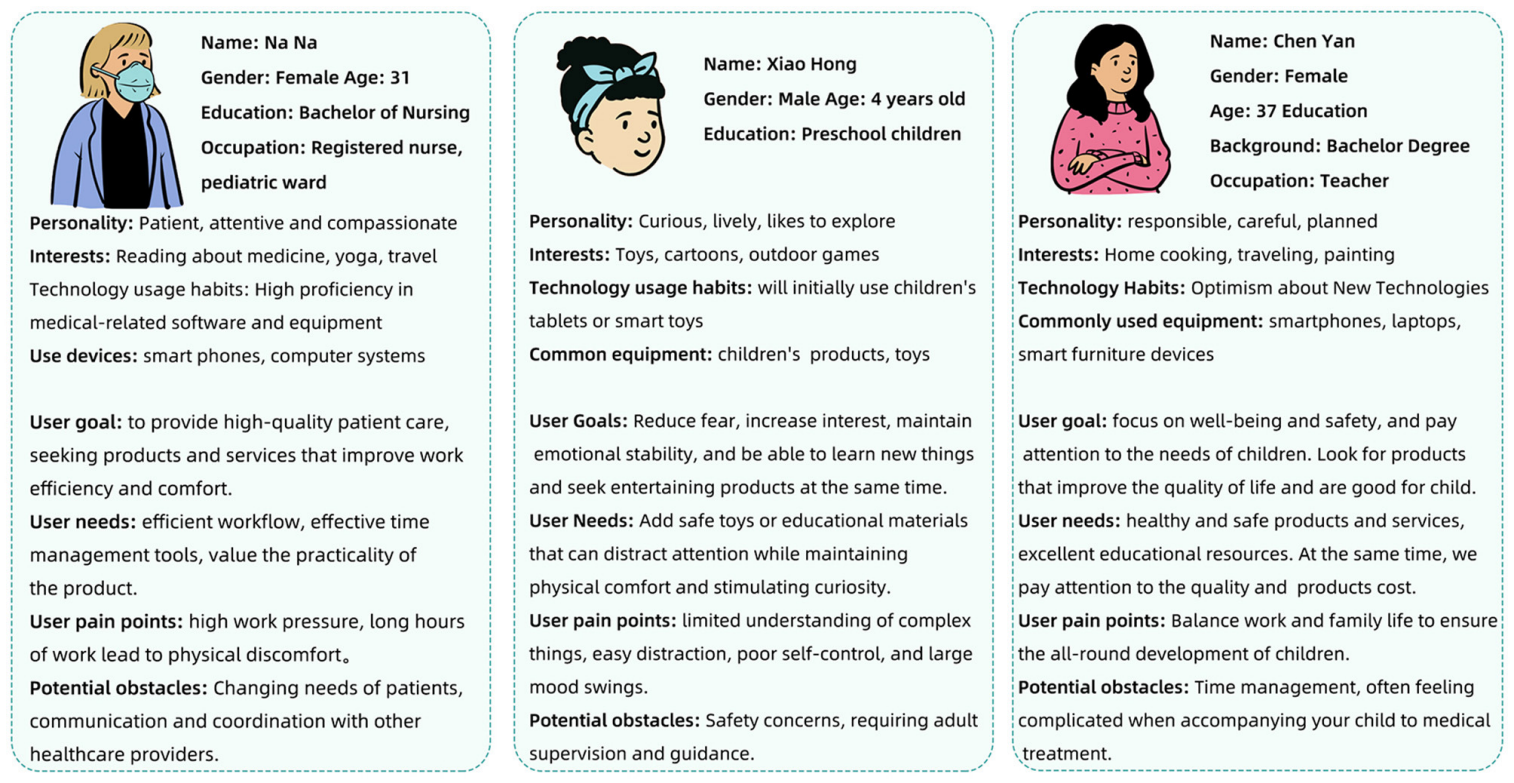
Children's medical product user portraits.

**Figure 5:**
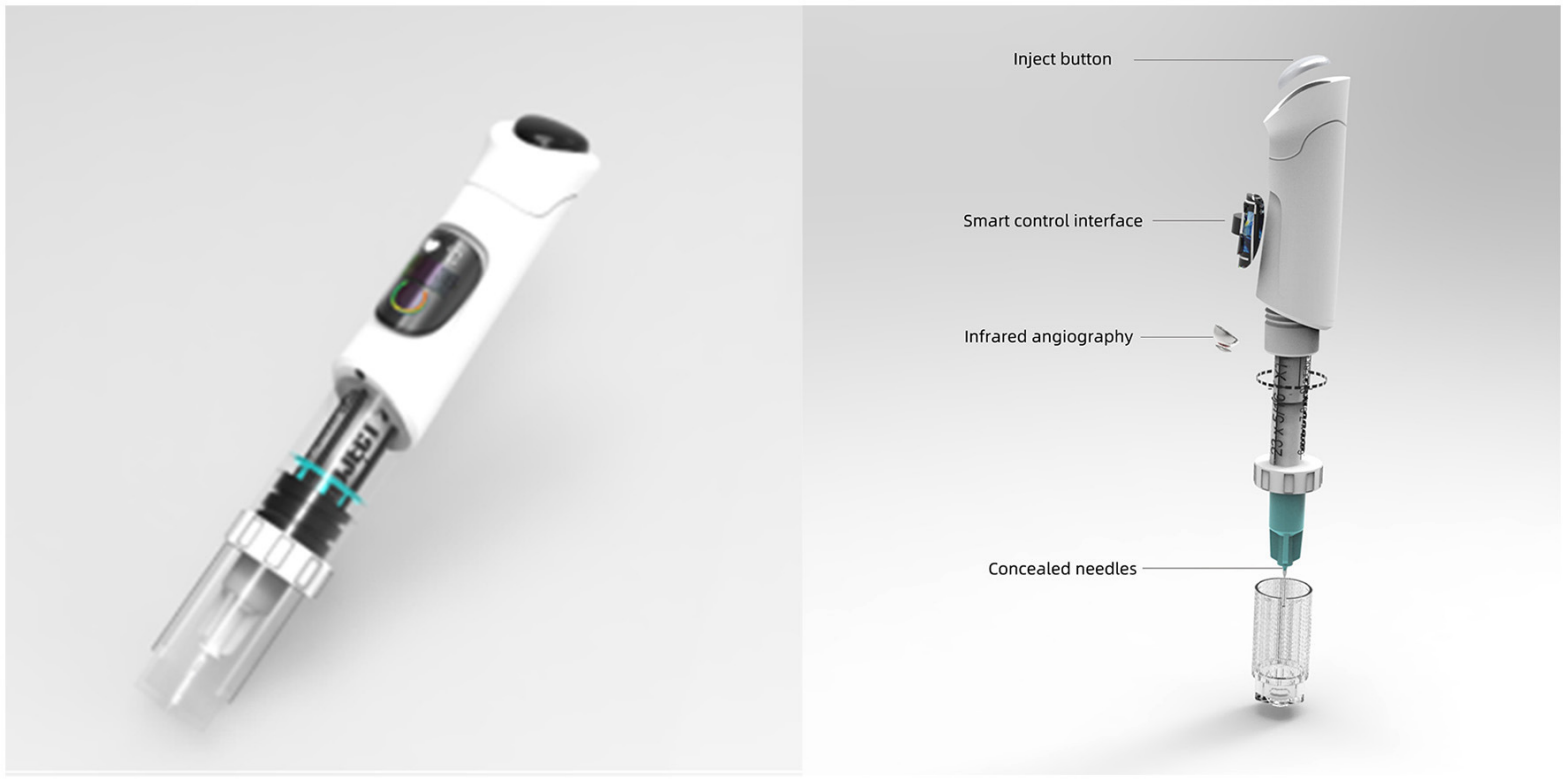
Children's medical syringe model diagram.

Additionally, we compiled a user profile based on feedback from children, healthcare professionals, and guardians, as illustrated in Figure 4. Utilizing this information, we employed computer 3D modeling to develop a conceptual model of a pediatric medical syringe, as shown in Figure 5. The model integrates features such as concealed needles, auto-injection, modular design, recyclability, real-time health monitoring, and angiography capabilities, and is designed to reduce anxiety and resistance in children during the injection process and enhance the treatment experience.

### 4.2 Sampling and participants

This study adopts a cross-sectional survey design, it is divided into online and offline questionnaire collection, offline we are in the pediatric department of Yichang Central Hospital, Hubei Province, we conducted a field study using a purposive sampling method, online distribution is carried out through the questionnaire platform “Wen juan xing,” recruiting parents of children aged 20–65 years in good health and without vision impairments like color blindness. Prior to the formal questionnaire, we conducted a pre-experiment study with two experts, two healthcare workers, and three parents, leading to revisions in perceived risk (PR), reduce time and error (RTE), and price value (PV) measures, as well as enhancements in the questioning techniques for other variables. The revised questionnaire is presented in Appendix A Table.

Human participants are included in this study, so all research methods are conducted in accordance with human-relevant ethical guidelines and regulations, including specific ethical frameworks. We obtained informed consent from all participants prior to their participation in the study. The experimental protocol was reviewed and approved by the Institutional Review Board (IRB) of Hubei University (Grant No.: 20230821).

**Figure 6:**
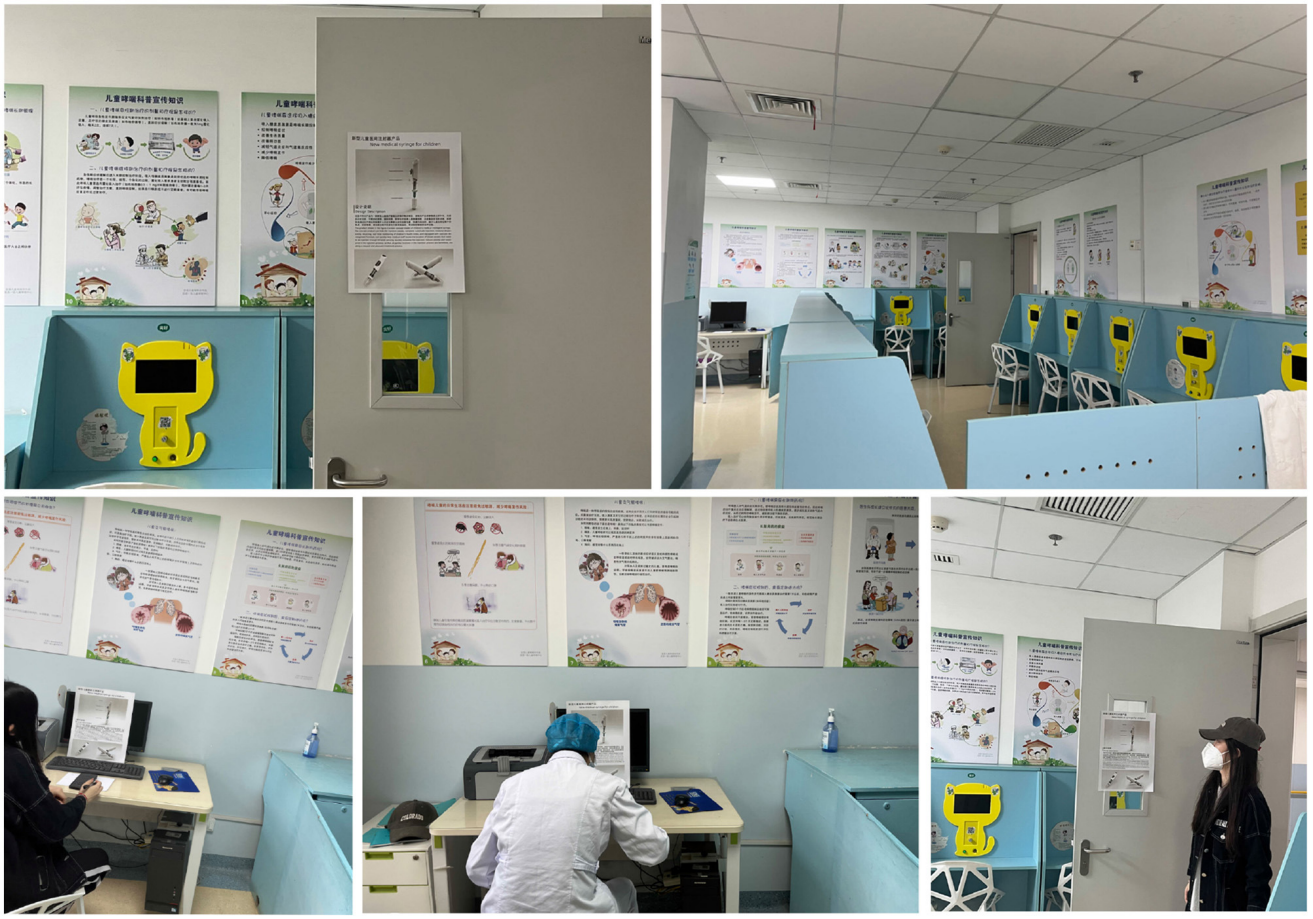
Field questionnaire collection.

Adhering to human research ethics, after giving users a detailed introduction to the structure and instructions for the use of the new pediatric medical syringe designed in this study, the participants successively filled in the online questionnaire, as shown in Figure 6. Subsequently, we clarified the study's objectives and procedures to participants, guaranteeing the confidentiality of their personal information. Participation was entirely voluntary, with confirmation available on the webpage before completing the questionnaire. Respondents could withdraw from the study at any time. A total of 463 questionnaires were received in this survey. After screening, eight invalid questionnaires (including five questions with abnormally fast answers, two questions with too consistent answers, and one with missing answers) were excluded, and finally, 455 valid questionnaires were obtained.

### 4.3 Measurement

The questionnaire, based on the TAM model and user requirements, consisted of three segments: the first stage presented the design and functionality of pediatric medical syringes. The subsequent section gathered basic details about the participants, including gender, age, and education, as well as their perceptions of pediatric medical syringes. Some questionnaire items were adapted from previous correlational studies, while others introduced new measures. The final section thanked the participants for their involvement in the survey.

Questionnaire responses were assessed using a 5-point Likert scale, where “1” represented strong disagreement and “5” represented strong consent. The Appendix A of the article contains the complete questionnaire as filled out by the participants. In the research model, hypotheses examined the impact of various constructs on behavioral intentions. The hypotheses posited that these constructs had a positive and significant effect on child caregivers' purchasing behavioral intentions (BI). The measurement of relevant variables utilized scales from existing studies, suitably adapted to fit the specifics of the healthcare industry.

## 5 Results

### 5.1 Descriptive statistics

We performed a characterization of the sample data using descriptive statistics, and the results are shown in Table 1. Statistical analyses were conducted using SPSS 22.0 software, and structural equation modeling (SEM) analyses employed a combination of Gaskin's plug-in, Excel worksheets, and AMOS 26.0 (Gaskin, 2016,?).

**Table 1:** Sample feature distribution.

**Statistical variables**	**Category**	**Frequency**	**Effective percentage**	**Cumulative percentage**
Gender	Male	130	28.6	28.6
	Female	325	71.4	100
Age	21–39	341	74.9	74.9
	40–59	98	21.5	96.5
	60 and above	16	3.5	100
Education level	Undergraduate or below	301	66.2	66.2
	Bachelor's degree	148	32.5	98.7
	Master's degree degrees	4	0.9	99.6
	Doctorate bachelor's degree	2	0.4	100
Children age	0–1	69	15.2	15.2
	2–3	74	16.3	31.4
	4–7	146	32.1	63.5
	8–12	166	36.5	100
Frequency of hospital visits	1–3/year	166	36.5	36.5
	1–3/ season	260	57.1	93.6
	Per month	29	6.4	100
Number of escorts	2–3	159	34.9	34.9
	1–2	296	65.1	100

### 5.2 Measurement model

Before conducting structural modeling tests, evaluating the measurement model is imperative. This study used a scaling approach for data collection, underscoring the importance of testing data quality to ensure the validity of subsequent analyses. Cronbach's alpha (Cronbach's coefficient) was utilized to conduct a rliability test, analyzing the internal consistency of the dimensions. Ideally, the CR value should exceed 0.7 (Barclay and Smith, 1995). As shown in Table 2, the Cronbach's alpha values all exceeded 0.8, indicating a very strong level of dependability.

**Table 2:** Internal consistency, reliability and convergent validity of the measurement model.

**Construct**	**Items**	**Estimate**	**CR (> 0.70)**	**AVE (> 0.50)**	**Cronbach's *a* (> 0.70)**
Perceived risk (PR)	PR1	0.769	0.840	0.567	0.839
	PR2	0.740			
	PR3	0.792			
	PR4	0.709			
Perceived ease-of-use (PEOU)	PEOU1	0.762	0.851	0.588	0.850
	PEOU2	0.783			
	PEOU3	0.775			
	PEOU4	0.747			
Perceived usefulness (PU)	PU1	0.799	0.849	0.584	0.848
	PU2	0.725			
	PU3	0.769			
	PU4	0.762			
Price value (PV)	PV1	0.739	0.813	0.521	0.811
	PV2	0.704			
	PV3	0.729			
	PV4	0.715			
Function (FUN)	FUN1	0.803	0.858	0.602	0.856
	FUN2	0.737			
	FUN3	0.815			
	FUN4	0.746			
Aesthetics (AES)	AES1	0.77	0.832	0.554	0.831
	AES2	0.712			
	AES3	0.750			
	AES4	0.744			
Reduce time and errors (RTE)	RTE1	0.736	0.838	0.563	0.837
	RTE2	0.763			
	RTE3	0.737			
	RTE4	0.766			
Technology anxiety (TAX)	TAX1	0.739	0.843	0.574	0.842
	TAX2	0.74			
	TAX3	0.776			
	TAX4	0.775			
Attitude toward use (ATU)	ATU1	0.800	0.882	0.651	0.882
	ATU2	0.779			
	ATU3	0.834			
	ATU4	0.813			
Behavioral intention (BI)	BI1	0.797	0.862	0.610	0.861
	BI2	0.749			
	BI3	0.792			
	BI4	0.785			

Subsequently, we determined convergence validity using composite reliability (CR), Cronbach's alpha, and extracted mean variance (AVE) according to the criteria created by Hair et al. (2021). AVE and CR values for the confirmatory factor analysis can be manually calculated with factor loadings and error variances from AMOS or Mplus, using the following formulas:

(1) AVE


(1) $$\begin{array}{l} AVE=\left(\Sigma \lambda^{2}\right)/n \end{array}$$

In Equation 1, λ: factor loadings, *n*: Number of measures of the factor

(2) CR


(2) $$\begin{array}{l} CR={\left(\Sigma \lambda \right)}^{2}/\left({\left(\Sigma \lambda \right)}^{2}+\Sigma \delta \right) \end{array}$$

In Equation 2, δ: Residual Variances, λ and δ are the result of standardization.

The analysis in Table 2 showed that AVE values of more than 0.5 and CR values of more than 0.8 for all dimensions of this validity test met the criteria for discriminant validity, convergence validity, and internal consistency in the development of the measurement approach (Barclay and Smith, 1995).

Furthermore, the relationships between variables in the diagonal matrix were analyzed (Fornell and Larcker, 1981), identifying each construct's AVE square root, with bolded figures surpassing the values in their corresponding rows and columns. This approach confirmed that the instrument demonstrates adequate discriminant validity, as illustrated in Table 3. Consequently, achieving convergent validity demonstrates that all dimensions have good convergent validity and composite reliability.

**Table 3:** Discriminative validity test form.

**Variables**	**PR**	**PEOU**	**PU**	**PV**	**FUN**	**AES**	**RTE**	**TAX**	**ATU**	**BI**
PR	**0.567**									
PEOU	0.544	**0.588**								
PU	0.390	0.520	**0.584**							
PV	0.403	0.506	0.325	**0.521**						
FUN	0.451	0.516	0.358	0.440	**0.602**					
AES	0.360	0.499	0.274	0.370	0.339	**0.554**				
RTE	0.400	0.502	0.416	0.329	0.372	0.382	**0.563**			
TAX	0.361	0.472	0.336	0.322	0.369	0.303	0.326	**0.574**		
ATU	0.367	0.500	0.393	0.353	0.401	0.273	0.344	0.337	**0.651**	
BI	0.398	0.545	0.388	0.383	0.439	0.285	0.409	0.371	0.373	**0.610**
AVE value square root	**0.753**	**0.767**	**0.764**	**0.722**	**0.776**	**0.744**	**0.75**	**0.758**	**0.807**	**0.781**

RTE, reduce time and errors; PR, perceived risk; TAX, Technology anxiety; PU, perceived usefulness; PEOU, perceived ease of use; PV, price value; AES, aesthetics; ATU, attitude toward use; FUN, function; BI, behavioral intention. The bold font refers to the AVE value of the corresponding variable.

Moreover, this study employed Pearson correlation analysis to explore the correlations between variables. The results presented in Table 4 indicate significant correlations between variables at the 99% significance level. The correlation coefficient r between variables being >0 indicates a significant positive correlation among the variables analyzed.

**Table 4:** Pearson correlation analysis between dimensions.

**Dimension**	**PRa**	**PEOUa**	**PUa**	**PVa**	**FUNa**	**AESa**	**RTEa**	**TAXa**	**ATUa**	**BIa**
PRa	1									
PEOUa	0.427**	1								
PUa	0.305**	0.426**	1							
PVa	0.307**	0.382**	0.228**	1						
FUNa	0.340**	0.404**	0.288**	0.308**	1					
AESa	0.294**	0.409**	0.211**	0.262**	0.291**	1				
RTEa	0.325**	0.384**	0.307**	0.227**	0.271**	0.302**	1			
TAXa	0.292**	0.410**	0.261**	0.230**	0.302**	0.218**	0.246**	1		
ATUa	0.279**	0.411**	0.311**	0.255**	0.323**	0.187**	0.287**	0.252**	1	
BIa	0.313**	0.429**	0.323**	0.290**	0.341**	0.177**	0.331**	0.312**	0.314**	1

** At the 0.01 level (two-tailed), the correlation is significant.

RTE, reduce time and errors; PR, perceived risk; TAX, technology anxiety; PU, perceived usefulness; PEOU, perceived ease of use; PV, price value; AES, aesthetics; ATU, attitude toward use; FUN, function; BI, behavioral intention.

### 5.3 Structural equation modeling

Before the assessment of the significance of the path coefficients in the structural model, we checked the model's consistency with the sample data. This verification process follows the methodologies outlined by Henseler et al. (2016). A goodness-of-fit (GOF) assessment was conducted to ensure the dependability and accuracy of the external model measurements, thus providing a valid evaluation of the internal structurally modeling (Soh et al., 2017). Using SPSS 26.0, multivariate normality was assessed, revealing no outliers or missing data in the dataset. Subsequent analyses used maximum likelihood estimation, given the data's normal distribution. The SEM software AMOS 26.0 was utilized for confirmatory factor analysis (CFA).

Table 5 indicates that the model's goodness-of-fit (GOF), as measured by the root mean square residual (SRMR) based on Henseler et al. (2016), has a threshold of < 0.08, with our study achieving an SRMR of 0.030. This result is in line with Hu and Bentler (1999) findings, which established a 0.08 threshold as suitable for new technology research using PLS path modeling. Therefore, the obtained SRMR value of 0.045 meets the criterion for model superiority, indicating a good fit.

**Table 5:** Model fit checklist.

**Norm**	**Reference standard**	**Data results**
CMIN/DF	Greater than 1 less than 3 is excellent, greater than 3 less than 5 is good	1.415
RMSEA	Less than 0.05 is excellent, and less than 0.08 is good	0.030
IFI	Greater than 0.9 is excellent, and greater than 0.8 is good	0.965
TLI	Greater than 0.9 is excellent, and greater than 0.8 is good	0.962
CFI	Greater than 0.9 is excellent, and greater than 0.8 is good	0.964

### 5.4 Results of path relationship hypothesis testing

**Figure 7:**
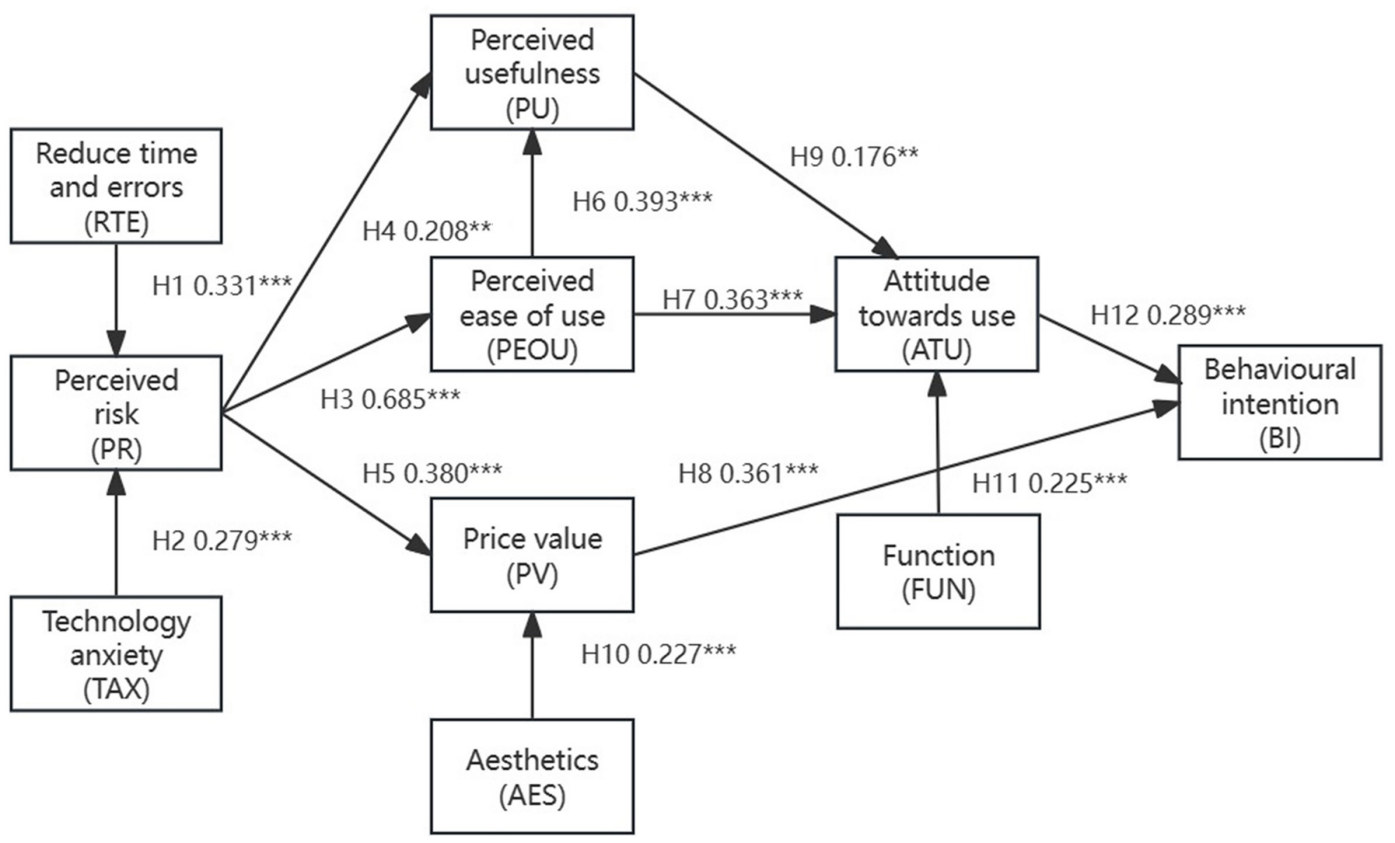
Path analysis results. ^**^p < 0.01, ^***^p < 0.001.

Structural Model (Path Analysis) depicts the assumptions about the relationships between the proposed model. Table 6 and Figure 7 detail the output data and results. All pathway effects were validated and their respective *t*-values were statistically meaningful, i.e., all hypotheses were supported.

**Table 6:** The result of the route relationship test.

**Hypothesis**	**Relationship**			**β**	**SE**	***T*-value**	***P*-value**	**Result**
H1	PR	< —	RTE	0.331	0.051	6.512	^***^	Support
H2	PR	< —	TAX	0.279	0.050	5.535	^***^	Support
H3	PEOU	< —	PR	0.685	0.070	9.836	^***^	Support
H4	PU	< —	PR	0.208	0.074	2.806	0.05	Support
H5	PV	< —	PR	0.380	0.059	6.464	^***^	Support
H6	PU	< —	PEOU	0.393	0.068	5.757	^***^	Support
H7	ATU	< —	PEOU	0.363	0.066	5.528	^***^	Support
H8	BI	< —	PV	0.361	0.065	5.585	^***^	Support
H9	ATU	< —	PU	0.176	0.065	2.719	0.07	Support
H10	PV	< —	AES	0.227	0.050	4.543	^***^	Support
H11	ATU	< —	FUN	0.225	0.053	4.222	^***^	Support
H12	BI	< —	ATU	0.289	0.051	5.647	^***^	Support

^***^p < 0.001.

β, path coefficients; SE, Standard Error; RTE, reduce time and errors; PR, perceived risk; TAX, technology anxiety; PU, perceived usefulness; PEOU, perceived ease of use; PV, price value; AES, aesthetics; ATU, attitude toward use; FUN, function; BI, behavioral intention.

The Technology Acceptance Model and its Technology Extension Model are frequently employed to evaluate multiple dimensions to understand the receptivity and usage of the technology by the target users. Radar charts offer an intuitive method for presenting multi-dimensional data, facilitating the rapid identification of key factors in the model and highlighting areas for further research or improvement. Consequently, we conducted a radar chart analysis on the basis of the datapaths shown on Figure 7. By analyzing the model roadmap, we identified hypothetical pathways and correlation coefficients among the model's components.

Moreover, we calculated the effect size *R*^2^ of the model, which ranged from 0 to 1, with larger values indicating greater explanatory power of the model to the data. A higher *R*^2^ value means that the model has higher prediction accuracy and a better fit. According to Cohen's (2016) effect size criteria, there are usually the following classifications: small effect: *R*^2^ = 0.02, medium effect: *R*^2^ = 0.13, and large effect *R*^2^ = 0.26.

In this research, *R*^2^ was calculated as follows: *R*^2^ of ATU was 0.281, BI was 0.216, PEOU was 0.371, PR was 0.313, PU was 0.294, and PV was 0.263. The above data show that 28.1% of the ATU changes can be explained by the PU, PEOU, and FUN independent variables in the model, and the effect size indicates that the model has a high degree of explanatory power for the ATU variables. 21.6% of the BI changes could be explained by ATU and PV independent variables, indicating that the model's effect on BI was at a moderate level. PR explained about 37.1% of PEOU changes, and the effect size was large, indicating that the model has strong explanatory power for PEOU variables. In addition, about 31.3% of the PR changes could be explained by the RTE and TAX independent variables in the model. 29.4% of the PU changes could be explained by the PEOU and PR independent variables in the model, and the effect size was at a high level. PR and AES explained 26.3% of the PV changes, and the effect size was also large.

Based on the comprehensive data results, most of the effect sizes of this research model are in the range of large effects, which is in line with the expectations of practical application. In particular, PEOU (perceived ease of use) and PR (perceived risk) had a large effect (*R*^2^ > 0.26), indicating that these variables have strong explanatory power for changes in the model. Therefore, from the perspective of effect size, the model of this study is of practical significance in general, especially the large effect size of PEOU and PR, which can better explain the changes in these variables.

**Figure 8:**
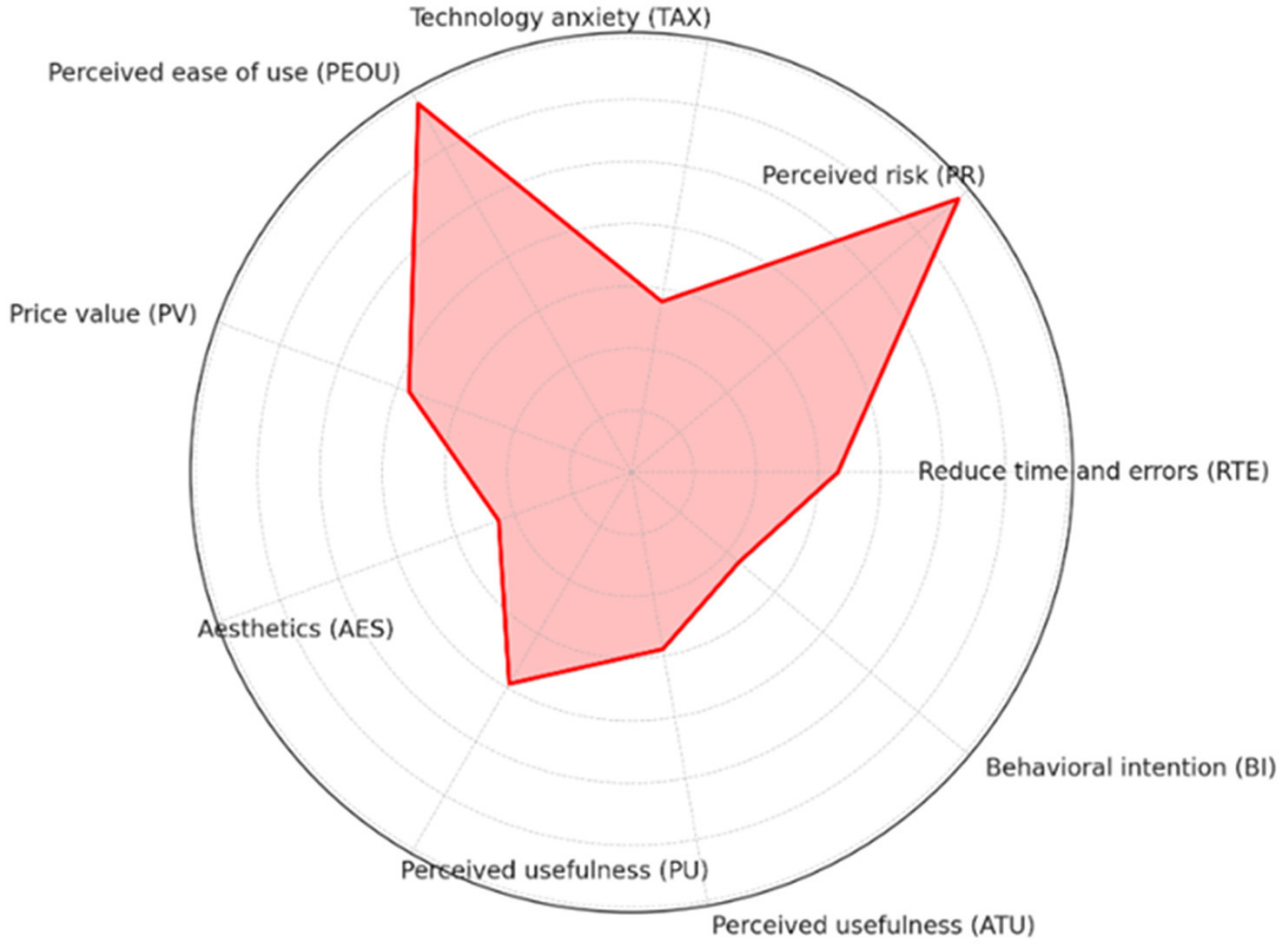
Numerical radar plot of model variables.

We utilized these coefficients to generate a radar chart, wherein path coefficients for each construct are depicted as points on the radar map, illustrating the values of each variable. The model's constructs are quantitatively evaluated by their maximum coefficients, with each axis representing a construct, and the axis length indicating the absolute value of the maximum path factor for that construct. Examination of the radar chart enables comparison of the constructs' relative importance within the model. Figure 8 clearly illustrates that the path coefficient from “PR” to “PEOU” stands at 0.685, the highest value, signifying The perception of risk substantial impact on perceived ease of use within this model. Conversely, the construct “aesthetics (AES)” exerts the least influence on other constructs, as evidenced by the shortest path coefficient.

**Figure 9:**
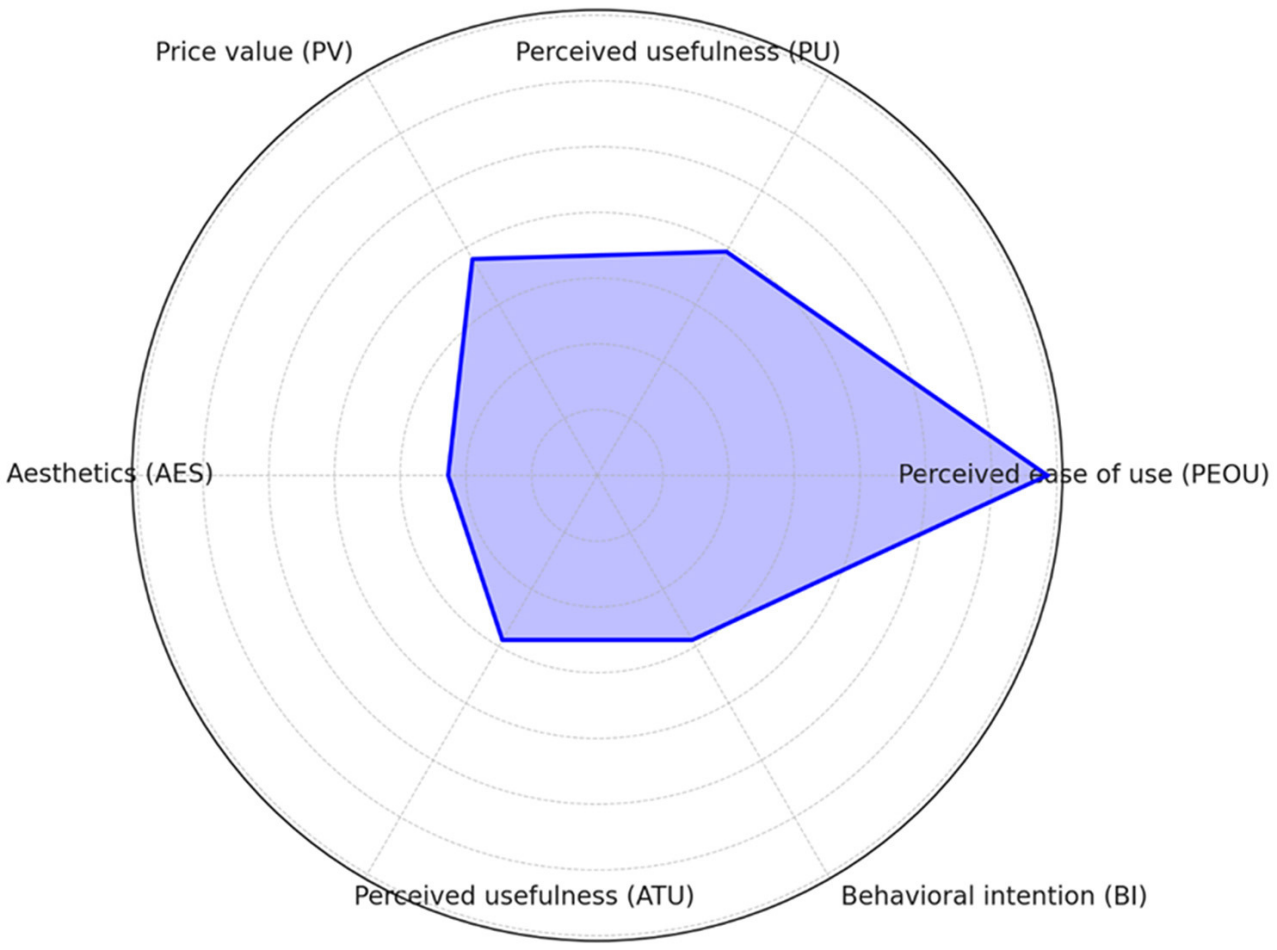
Radar plot of the model path coefficients.

Furthermore, to demonstrate the relative importance of each construct in the path model, We focus on using the absolute value of the coefficients as a scale to show the endogenous structures in the model, where the radar axis of each structure reflects its relative importance in the model based on the absolute value of the coefficients. Figure 9 vividly demonstrates that “PEOU” is the most significantly prominent construct, as it boasts the largest path coefficient from “PR” to “PEOU,” underscoring its paramount importance in the model. The significance of other constructs is gauged by the length of their corresponding axes, where a longer axis denotes greater relative importance.

## 6 Discussion and implications

### 6.1 Discussion

This research examines factors influencing user acceptance of children's medical supplies and guides for developers to enhance this acceptance and satisfaction. To accomplish this objective, the TAM model was expanded to include new variables. As per the findings of this research, the ensuing strategies are proposed to enhance the market for children's medical supplies:

First, the noticeable effect of PEOU and PU on children's medical products suggests designs should be user-friendly and straightforward for children or users to operate.

Second, products' functionality and aesthetics should be tailored to children's unique characteristics, age, and size. Designs must prioritize safety and comfort, incorporating non-toxic materials and avoiding sharp edges.

Third, the pronounced effect of price value on consumer behavioral intentions underscores the criticality of cost and quality in purchasing decisions. Parents might be willing to pay a premium for high-quality, dependable children's medical products.

Fourth, perceived risk markedly impacts price, usability, and utility, denoting users' heightened risk sensitivity in the context of children's medical products. Users demonstrate a pronounced risk sensitivity when it comes to children's medical products. This suggests that perceived risk shapes users' attitudes toward new technologies, thereby indirectly influencing technology acceptance. Recommendations by healthcare professionals and the adoption of new technologies in children's medical supplies can alleviate consumer hesitation.

Fifth, technology anxiety and error reduction exert a direct impact on perceived risk, potentially resulting in extended user skepticism due to safety concerns. Consequently, a principal challenge for designers and suppliers of children's medical products lies in earning user trust and ensuring product safety to reduce perceived risk. Moreover, initiatives should be undertaken to popularize new children's medical technologies, aiming for wider acceptance.

Finally, the study found that the explanatory power of aesthetics on price sensitivity is still weak (H10 PV← AES, β= 0.227), which may be due to the fact that consumers tend to focus more on functionality (e.g., safety, practicality, and ease of use) than aesthetic design when evaluating medical products, so the AES path coefficient is given a lower priority in the overall decision-making framework. In addition, in a given cultural context (e.g., China), the aesthetic characteristics of medical products may not have as significant an impact on price acceptance as other products (e.g., fashion or consumer electronics). Consumers often make purchases based on product performance, medical value, or expert recommendations, rather than aesthetics. While aesthetics can enhance product appeal, consumers are more likely to consider price comparison, functional value, and brand credibility when it comes to price sensitivity. This suggests that while aesthetics play an important role in attracting consumers' attention and influencing their initial interest, price factors and functional factors may be more decisive in actual purchasing decisions.

The extended Technology Acceptance Model (TAM)-based analysis facilitates a deeper comprehension and refinement of the design and promotional strategies for children's medical products, ensuring closer alignment with the needs and expectations of children and their caregivers.

### 6.2 Theoretical contribution

#### 6.2.1 Cognitive processes

The central objective of this investigation was to examine how diverse factors within cognitive processes affect consumer attitudes toward children's medical supplies. This study demonstrated that PEOU and PU substantially influence consumer attitudes. Simplicity, intuitiveness, and comprehensibility are crucial factors in children's medical products. If users perceive a product as easy to use, their likelihood to accept and adopt it increases. Furthermore, users need to acknowledge the positive effects these products can impart on children's health, encompassing their efficacy in treatment, monitoring, and maintenance. Consequently, within the context of kid's medical products and grounded in the TAM model, this study sought to identify methods to bolster parental and healthcare professional acceptance and usage, facilitating market promotion and popularization, and allowing consumers to develop initial perceptions vital for market expansion.

#### 6.2.2 Design process

The research's second objective focused on the design elements of form and function, utilizing Aesthetics (AES) and Functionality (FUN) to investigate their influences on the design of children's medical supplies. This study found that the essential design elements are grounded in the User-Centered Design (UCD) concept. Designers ought to consider emotional factors that influence consumer purchasing attitudes, encompassing children's physical and psychological characteristics, values, and the needs of healthcare professionals. The design process must involve engagement with potential users to facilitate iterative product optimization, including prototype testing, gathering feedback from customers, and adjustments based on market demand. Consequently, the design elements, encapsulated as FUN and AES, are refined through a design science methodology, concentrating on fundamental user needs to guarantee the product conforms to public aesthetic standards and retains its versatility.

#### 6.2.3 Design-making process

The third aim was to investigate how perceived risk (PR) and price value (PV) influence consumer attitudes in selecting children's medical products. Findings indicate that PV directly and significantly impacts attitudes toward usage (ATU), whereas PR indirectly affects ATU via PEOU, PU, and PV. Concerning PV, cost-benefit analyses demonstrated that both parents and healthcare organizations consider the cost-benefit ratio in their decision-making, suggesting the product must be medically effective and economically viable according to market standards. PR complexity surpasses that of PV, as decisions by parents and healthcare providers may be influenced by societal attitudes and professional opinions. Understanding these factors is essential for product acceptance. This is consistent with prior work, which indicates that understanding end-user sentiment toward technology is critical to building consumer trust and overcoming obstacles to innovation (Ahmad et al., 2020).

### 6.3 Management implications

In addition, our discoveries point the way for managers to when considering the launch of relevant novel pediatric medical products. Firstly, companies must adopt effective marketing and promotional strategies to encourage target users to embrace their products. Secondly, offering incentives like discounts and rewards can enhance users' willingness to adopt the products. At the same time, engaging users in product design and feedback processes can significantly increase their engagement and adoption willingness. Finally, creating a positive brand image via marketing strategies can further enhance users' willingness to utilize these products. Implementing these management strategies will improve market acceptance and usage of children's medical products, ensuring their safety and effectiveness for children. These management practices not only contribute to the product's market success but also play a crucial role in improving the healthcare system's overall quality and efficiency and the sector's development. The specific measures are as follows:

(1) Simplify product operations: companies in the children's medical product industry should prioritize enhancing product intuitiveness and user-friendliness to streamline operations. Moreover, companies must provide comprehensive user training, concise instructions, and effective technical and customer support systems to quickly resolve user issues. By implementing marketing and social awareness campaigns, enterprises can boost public acceptance of children's medical products and promote them through partnerships with medical and educational institutions.

(2) Develop comprehensive user feedback processes: product effectiveness must be validated through scientific research and case studies, illustrating its contribution to improving children's health and care quality. Furthermore, companies should utilize user feedback to enhance communication and collaboration among R&D, sales, customer service, and other departments, ensuring optimal resource allocation (capital, manpower, time) to boost the company's credibility and professionalism.

(3) Minimize potential product risks: enterprises must ensure all products comply with industry standards and regulatory requirements, ensure their products adhere to safety standards to minimize potential risks and effectively communicate product safety and risks to users. Moreover, companies should address users' fears and anxieties regarding the technology with supportive interactions and positive communication strategies. Implementing these risk mitigation measures contributes to the long-term stability, success, and sustainability of the products, enhancing the concern's professionalism and establishing a robust brand reputation in the industry.

(4) Enhance and innovate product functionality and design: while fulfilling the basic functional requirements of children's medical products, enterprises should continuously innovate product features to keep pace with market trends and user needs. Additionally, the design must be child-friendly and cater to users' aesthetic preferences. Enterprises should prioritize user experience, emphasizing emotional and sensory aspects to enhance product appeal and boost brand competitiveness.

(5) Price according to market expectations: considering the need for advanced technical support and stringent material selection in medical supplies, the associated costs are typically high. As a result, companies should highlight the long-term advantages of their products when devising a pricing strategy that appeals to a wide array of users. This strategy involves setting fair prices and demonstrating the product's value via marketing efforts to ensure users recognize the rationale behind their long-term investment.

### 6.4 Limitations and future research

There are some limitations in this study, firstly, because this study adopts a cross-sectional method for design research, there are certain limitations in the inference of causality, and future related studies can further verify the research conclusions between variables through longitudinal data research or experimental research, and can further provide dynamic changes in the time dimension of user attitudes of the same product. On the other hand, the data in this study are mainly derived from questionnaire methods, which may be at risk of common methodological bias, and future studies can control and validate through multi-source data or experimental design. Most importantly, cultural factors are likely to influence the results of the study, for example, Chinese parents' perception of new medical products for children may be influenced by more conservative attitudes toward medical technology, which may limit the generalizability of the research conclusions. Therefore, future research could further explore how cultural factors affect the acceptance of medical products for children.

As the data in this study was solely obtained from China, and variations in the gender of child guardians across different countries and cultures may influence the usage patterns of children's medical supplies. Therefore, future work is required to determine the applicability of the proposed model across various cultural contexts.

At present, awareness of new children's medical products among Chinese parents is relatively limited. The model and findings from this study could be instrumental in developing children's medical products that cater to diverse needs and in furthering research on relevant marketing strategies and usage intentions. Considering the swift advancements in the medical and healthcare industry, new smart medical products are expected to become increasingly popular. The distinctive features of medical devices for children provide an opportunity to understand the factors driving their acceptance and usage, facilitating more efficient system design in the pre-production phase. Product design informed by this study's model is poised to advance the children's medical industry and potentially has broader applications for adults, pregnant women, and the elderly.

## 7 Conclusion

The purpose of this study was to explore the acceptance of children's medical syringes by parents and to extend multiple relevant variables based on the TAM model. The new variables include product characteristics (RTE, reduce time and errors; PR, perceived risk; TAX, technology anxiety) for pediatric medical syringes and common product evaluation factors (e.g., price-value, aesthetics, functionality) used by users. The results show that all paths in the model are supported. Data path analysis found that PEOU (Perceived ease of use) and PU (Perceived usefulness) had a significant impact on children's medical products. The functionality and aesthetics of medical products need to be tailored to the individual differences of children, but safety should be a primary consideration. Price value plays an important role in users' purchase decisions, and perceived risk significantly affects the relationship between price value and usefulness. The characteristics of technology anxiety and reduce time and errors have a direct impact on perceived risk. While aesthetics can attract initial attention, the final purchase decision is more influenced by price, value, and functionality. Although the study provides valuable insights into Chinese parents' acceptance of children's medical products, there are some limitations, such as the cross-sectional design limits causal inference, and future studies can use longitudinal data or experimental design to verify variable relationships and reduce methodological bias. At the same time, the influence of cultural factors on the attitudes of parents of children cannot be ignored, such as the conservative attitude of Chinese parents toward new medical technologies, and future research can explore the impact of cross-cultural differences on the user acceptance of medical products. Given the rapid development of the medical industry, the findings of this study will help design products that are more responsive to the needs of children, and these findings can also be extended to product design for other groups such as adults, pregnant women, and the elderly.

## Data Availability

The raw data supporting the conclusions of this article will be made available by the authors, without undue reservation.
