# Supplementary material for: Parental acceptance of novel children's medical syringes and their influencing factors
Source: Front Psychol. 2025 Mar 11;16:1454108. doi: 10.3389/fpsyg.2025.1454108 (PMC11932982; doi:10.3389/fpsyg.2025.1454108)
Supplement: Supplementary file 1 [file Table_1.docx]

**Appendix**

**Table A** Measurement items of the questionnaire

| **Construct** | **Items** | **Survey Item** | **References** |
| --- | --- | --- | --- |
| Perceived risk (PR) | PR1 | You are concerned about the safety performance of children's medical syringes. | Park et al. (2019) |
|  | PR2 | You are worried about whether the product will work satisfactorily. |  |
|  | PR3 | You are worried that using the product is not technically mature enough. |  |
|  | PR4 | You are worried about whether the product matches your subjective impression and identity. |  |
| Perceived ease of use  (PEOU) | PEOU1 | You think learning how to use the product is easy for healthcare workers. | Kwangsawad and Jattamart (2022) |
|  | PEOU2 | You don't think it's difficult to use the product. |  |
|  | PEOU3 | You don't think a lot of mental work is required to use the product. |  |
|  | PEOU4 | Overall, you think the product is easy to use. |  |
| Perceived usefulness  (PU) | PU1 | The use of this product can improve the work efficiency and quality of medical staff. | Kwangsawad and Jattamart (2022) |
|  | PU2 | The use of this product will improve the comfort of the child during the injection and make the medical treatment process easier and more enjoyable. |  |
|  | PU3 | The use of this product can reduce children's anxiety and resistance, thereby reducing the injection error rate. |  |
|  | PU4 | All in all, the use of this product is beneficial for healthcare professionals and children. |  |
| Price value | PV1 | The product offers good value at current prices. | Natarajan et al. (2017) |
| (PV) | PV2 | You think it's important that the product price matches the value (value for money) |  |
|  | PV3 | If you use the product more than the estimated cost, it will affect your intention to purchase/use. |  |
|  | PV4 | Using the product was worth it for me compared to the time spent. |  |
| Function  (FUN) | FUN1 | Using the product was worth it for me compared to the time spent. | Wang et al. (2021) |
|  | FUN2 | Using this product is a good way to get the injection in children. |  |
|  | FUN3 | This product can monitor children's health status in real time to ensure their safety. |  |
|  | FUN4 | The functional performance of the product is stable. |  |
| Aesthetics  (AES) | AES1 | Whether the product is a favorite color choice for children. | Malmivaara et al. (2009) |
|  | AES2 | Whether the product is desirable for children. |  |
|  | AES3 | Whether the product is a material that children like. |  |
|  | AES4 | Do you like the professionalism of the appearance of the product. |  |
| Reduce time and errors  (RTE) | RTE1 | Using this product can help healthcare professionals reduce injection errors and time during injections. | Chen et al. (2023) |
|  | RTE2 | Using this product can reduce the time required to find the child's blood vessels and quickly locate the location of the injected blood vessels |  |
|  | RTE3 | Using this product can help guardians reduce their time on the event. |  |
|  | RTE4 | The use of this product can reduce injection errors caused by children's anxiety during injections. |  |
| Technology anxiety  (TAX) | TAX1 | There is general anxiety about the technical reasons for a new product and a fear of making mistakes. | Park et al. (2019) |
|  | TAX2 | The difference between the product and what you expect can make you anxious. |  |
|  | TAX3 | Are you ambivalent about the product (want to try it and worry). |  |
|  | TAX4 | Anxiety about the usage of the product that needs to be learned. |  |
| Attitude towards use  (ATU) | ATU1 | You are willing to know the details of the product. | Shaw et al. (2003) |
|  | ATU2 | You are willing to try the product. |  |
|  | ATU3 | It is good for children to use the product. |  |
|  | ATU4 | Your attitude towards the product is positive. |  |
| Behavioural intention  (BI) | BI1 | All things considered, you'll want to use the product. | Venkatesh et al. (2008) |
|  | BI2 | You'll buy the product when you need to use it. |  |
|  | BI3 | I will use it often in the future if I have the opportunity. |  |
|  | BI4 | You will recommend the product to those around you. |  |
